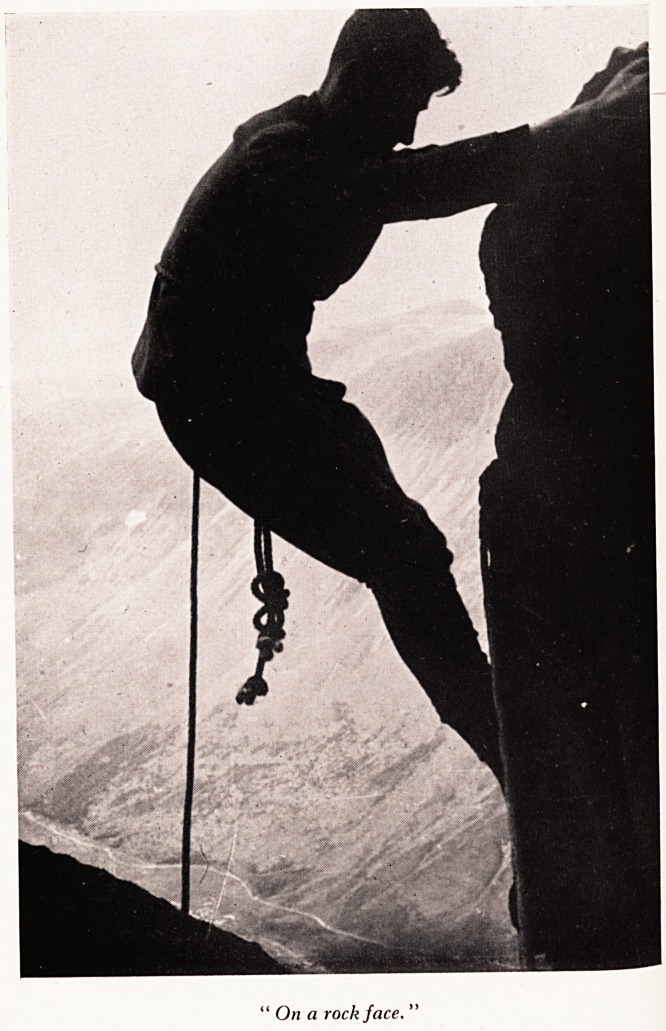# Mountains
*Presidential Address to the Bristol Medico-Chirurgical Society, Wednesday, 14th October, 1959.


**Published:** 1960-07

**Authors:** F. W. Terrell Hughes


### MOUNTAINS*

BY

DR. F. W. TERRELL HUGHES

I am a general practitioner in a rural practice. Perhaps the peace and quiet of the country tend to make one think more slowly and one is less likely to impute ulterior motives. When I was asked to become a member of the Med-Chi Committee, I thought no farther than that they perhaps wanted an elderly practitioner to represent that part of Somerset that lies on the southern boundary of Bristol. When I was later asked if I was willing to be elected President of this honourable and learned Society, my rustic mind received a shock. My first impulse was to refuse. I then remembered I was a G.P. and if I accepted I was being, so I thought, a representative of my G.P. colleagues and to refuse would, in a way, be letting them down. That is why I am here tonight, very conscious of this high honour bestowed on me by my colleagues in all the branches of medicine. Tonight I have roused myself from my rural relaxation, combed the straws from my hair and with the help of my friend at the epidiascope, hope to keep you interested in this talk about mountains.

As I began to plan this talk about mountains, I found there was much that could be related to medicine. Perhaps in general practice more than in any other branch of medicine, one is brought closer to the personality and environment of the patient and a consideration of these two things can be of help in his treatment.

Among mountains personality and environment matter a great deal and I have been often conscious of the personality of a mountain. To put it very simply, I have climbed one mountain that I have felt was always friendly, even though its moods might vary, and I have climbed another that has had to be tackled with greater care because it was unfriendly.

### WHY CLIMB ?

No mountaineer when asked has been able satisfactorily to answer the question, Why do you climb? No one can explain to someone who has not felt it what a belief really feels like, nor if you have felt a belief is there any need to have it explained to you. Practice can often be defined in concrete terms, but motives can rarely be defined.

In the course of my experience in thinking, reading, and climbing, I have found two definitions of mountaineering. (1) Mountaineering is an unconscious search for beauty. (2) Mountaineering is the art of going safely in dangerous places. I had intended to discuss these two definitions separately, but as I wrote I found that one became involved with the other, interwoven and inseparable, and if I have given proper expression to my thoughts you will see how both of these definitions apply to true mountaineering. The second definition—"the art of going safely in dangerous places" could be applied to other activities as no doubt my surgical colleagues would agree, though theirs is a field in which I personally could not roam in safety. To go back to my first definition, the search for beauty, I do not mean we say "I must go and look for beauty on a mountain". It is rather that at some moment through some conscious act, one unexpectedly experiences a feeling of peace and relaxation, of achievement and even elation. This is experienced in art, music, philosophy and in religion, and Beauty is its name.

Painters cannot explain the need to paint and I believe an attempt to explain would end in a failure to paint. Given time and sufficient discernment laws could be

*Presidential Address to the Bristol Medico-Chirurgical Society, Wednesday, 14th October, 1959.

2

found to explain why a mountain always looks right and a painting or a photograph of a mountain sometimes looks wrong. The early masters rarely painted mountains. It is known that people looked upon mountains as places of dread and horror and painters and poets generally reflect the ideas and impressions of their time; they may be a little ahead of their time but they are influenced by mass suggestion. Wordsworth was one of the early romantics; he is not often read or quoted today but he was one of the first poets to see beauty in all nature and he was reflecting the thoughts of the people of his generation. They were beginning to see beauty and not fear in the higher and more remote parts of the earth.

There is also the inherited fear of mountains which was handed down and persisted through the Middle Ages. It is interesting to realize how this has become a thing of the past. It still persisted towards the end of the eighteenth century and was almost entirely dispelled by the inquiring mind of a scientist, in the broad sense of the word. He made science his excuse for climbing. In 1786 Dr. Paccard climbed Mont Blanc, justifying his efforts by the necessity to enquire into and make unprejudiced observations on conditions at this altitude. Others followed his example, nominally in the cause of science, and they were largely instrumental in ridding mountains of their myths and monsters. Yet there are still records of climbers who have felt the presence of another person in their team. Frank Smythe on one occasion divided his food ration into two parts and turned to hand one portion to the presence he felt beside him but found was not there. I think it likely that fatigue, lack of oxygen, and the tension of the situation explain this, but I can assure you the hallucination is very real.

Those who follow the ways of the hills are still often objects of astonishment. You may be asked if it is a question of heredity and when you say, "No, my father and my grandfather did not climb", the questioner turns away and sadly shakes his head, but you still go and climb hills. As you get away into the hills, you get away from mass suggestion and as you become aware of this freedom, relaxation becomes possible, and so your thoughts can take shape, influenced only by nature and its high hills.

As you climb you have to make decisions which involve an estimation of the strength of the mountains, you learn your limitations and know the mountain is always the stronger. You learn to make an effort to attain and you can share this exhilaration with those who climb with you. Much is said of the value of team games but, though mountaineering is not a game, I know of no team spirit that can compare with that of climbers, tied to a rope on a rock face. Whenever I was leading a climb with my brother second on the rope, he always knew if my hesitation before making a move was to decide how it could be best done or because I was anxious that I could not make it at all.

Fear must be respected on a mountain. I remember in my early climbing days being repeatedly told, if you become frightened, turn back. I do not think I have indulged in what may be called dangerous sports, and I believe than an analysis of the motives of those who have done so would show that they are not undertaken because they are dangerous. It is the challenge to achieve and overcome difficulties. I am absolutely certain that the real mountaineer does not climb for the fun of doing something dangerous. Thinking back to my early days of climbing and those men who taught me what I know of climbing, the first, second, and third lesson was always the technique and principle of climbing safely. It is the principle of always endeavouring to climb with a margin of safety, sometimes a very small margin, that produces the confidence that makes achievement possible even in great difficulties. One might say that perfect confidence casteth out fear. May I give you an incident in which, thinking back as I have many times, I am sure I had no fear and every confidence. Six of us were climbing in Switzerland. On my rope was a guide leading, then a girl, and myself third. On the other rope were three expert and experienced climbers. We were going up a steep snow slope and our rope was some way ahead of the other when we came to a crevasse. This crevasse was about fifteen feet across, and it was spanned by a snow bridge; that is, frozen snow had left a possible means of crossing and the bridge was about three feet wide. I pushed my ice axe well into the snow and played out the rope as the guide went over on all fours, to distribute his weight. I saw the bridge shake and tremble a little during his passage. He cut steps up the steep slope on the far side and put his ice axe well into the snow and called to the girl to follow. She was safeguarded both by my belay and the guide's. Again I watched an even greater shaking of the snow bridge, but she made the crossing safely and, on the far slope, made herself secure with a belay around her ice axe. The guide called to me to come on. I told him the snow bridge would not take my weight, and for a few minutes we shouted to each other, he in his broken English and I in my broken French and then I gave in, and going on all fours began my passage along the bridge, my axe slung by its loop to my wrist. As I expected, when just over half way across, the bridge gave way. Being prepared for this I did my best to fling myself on to the far side, and the pull of the guide's rope enabled me to land with my arms on the slope and my legs dangling in the crevasse. It was a wonderful experience, first to hear the débris falling and never to hear it reach the bottom, and then to look down into the green depths of the crevasse. I then took the point of my ice axe, and digging it into the soft snow on the edge endeavoured to get a grip to pull myself out. Every time the steel point came through the soft snow and I could get no grip. I felt perfectly safe and had no feeling of alarm or fear at all. The two above me could not move from their belays as they were holding me, and I wondered what was the next thing to do. The smooth hard ice of the crevasse gave no grip for my nailed boots. I rested a moment and then heard a voice behind me say "Turn your ice axe round". I looked over my shoulder and saw the three experts on the other rope standing watching and grinning at my futile efforts. I turned my ice axe round, drove the blade instead of the point into the snow, obtained a hold and was soon out of the crevasse. It was a lesson learnt in technique, but at no time did I have any feeling of fear for the outcome.

If fear is deliberately and continually incurred, as it can be, it destroys those feelings that mountaineers value so highly.

### FALLS

As regards a fall, it seems to be true that after the initial shock of losing balance or coming off a hold, fear is generally absent during the act of falling. There is a feeling of detachment. There are fanatics who say there is actual pleasure experienced during the act of falling. I do not know, for it is one of the pleasures I have never been in a position to enjoy; but I can give you the account of the distinguished and experienced mountaineer, Frank Smythe, who has recorded his thoughts after a fall*. He was climbing in the High Alps and he says it was due to his carelessness that the fall took place.

"When I heard Roberts's shout and the crash of falling rocks, my body instinctively braced itself to receive a shock. The shock came; I was unable to resist it, and found myself on my back sliding and bumping helplessly down the slabs of the ridge. Now, one half of my brain must have known subconsciously that twenty feet of slack rope separated me from the belay, but if it did know this, it was singularly reticent on the subject, and it was the other half that took charge, and this told me that I had been secured close to the belay, that the rope had come off and that I was certain to be killed. In view of my subsequent sensations, the certainty which existed in my mind that nothing could stop my falling and that I was to be killed, is interesting and

*From Frank Smythe's *The Spirit of the Hills*. Reproduced by kind permission of the author's widow, The Viscountess Molden, and of the publishers Messrs. Hodder and Stoughton, Ltd.

important. Nevertheless, even though I had assumed thus early that I was as good as dead, I made desperate attempts to stop myself, as I have already described. During the time that I was doing this, a curious rigidity or tension gripped my whole mental and physical being. So great was this tension that it swamped all pain and fear, and rendered me insensible to bumps and blows. It was an overwhelming sensation, and quite outside my experience. It was as though all life's forces were in process of undergoing some fundamental evolutionary change, the change called death, which is normally beyond imagination and outside the range of ordinary human force or power. I felt that power which alone can separate spirit from body—death. I know now that death is not to be feared, it is a supreme experience, the climax, not the anti-climax of life." (We who see people near death should find his opinion significant.)

"For how long I experienced this crescendo of power I cannot say. Time no longer existed as time; it was replaced by a sequence of events from which time as a quantity or quality in terms of human consciousness no longer existed. Then, suddenly, this feeling was superseded by a feeling of complete indifference and detachment, indifference to what happened to my body, detachment from what was happening or likely to happen to that body. I seemed to stand aside from my body. I was not falling, for the reason that I was not in a dimension where it was possible to fall. I, that is my consciousness, was apart from my body, and not in the least concerned with what was befalling it. My body was in the process of being injured, crushed and pulped, and my consciousness was not associated with these physical injuries, and was completely uninterested in them. Had the tenant already departed in anticipation of the wreck that was to follow? Had the assumption of death—when my slide was not checked by the rope I assumed death as certain—resulted in a partial dissolution of the spiritual and physical? Was it merely a mental effect due to a sudden and intense nervous strain? It is not within my province to discuss that which only death can prove; yet to me this experience was a convincing one; it convinced me that consciousness survives beyond the grave.

"Had I died and passed on to another plane, I might have carried one memory with me. As I shot down the slabs and out over the edge of the sheer precipice, I saw stretched before me and below me the far-reaching, level, sunlit ocean of cloud, out of which we had climbed; I seemed to be diving straight into it. Above these clouds the sky was unclouded, a deep, profound blue from which the sun shone with dazzling radiance. I should have taken with me a memory that a king might have envied.

"Then I found myself hanging on the rope. The fact that I can remember no shock, and that I was not stunned, for my head received no blow, is interesting, as it bears out what I have already written on the feeling of detachment that was a feature of the fall. It was as though I was brutally dragged back to earth and earthly sensations. Simultaneously with the realization that I had not been killed, that something had arrested my fall, came fear. It was a curious, paradoxical thing to happen. During the fall I was not afraid, and now the fall had been stopped I was desperately afraid. I clutched and clawed at the rocks, and gained a footing and a hand-hold. I paused for a moment to try to still the thumping of my heart and the clamour of my lungs, which had been squeezed in by the pressure of the rope. Then I climbed, somehow or other, up to the ridge."

### THE MOUNTAIN RESCUE ORGANIZATION

If you take the road from Capel Curig in North Wales, towards Snowdon and the Llanberis Pass, you come upon a delightful building which is the Pen-y-Gwyrd Hotel. From here, under the leadership of Christopher Briggs, goes out the Mountain Rescue Organization, when it is called upon to help those who have been unfortunate enough to have had an accident on the mountain.

*"'Climbing' in a rock chimney."*

*" On a rock face."*

Many accidents are avoidable and learners cannot be too particular in observing and acquiring the technique their leaders are so willing to teach. One cannot afford to make mistakes when climbing, and avoidable accidents bring disrepute to one of the finest enterprises men and women have undertaken since the eighteenth century. A climb that is within the scope of a moderate climber in good weather may test the skill of the most experienced if the weather changes. When setting off climbers should always leave information of the mountain and climb to be attempted. The members of the Rescue Party are devoted and experienced climbers with an intimate knowledge of the mountains of Snowdonia and will always endeavour to bring an injured climber off the mountain to the road where the ambulance is waiting. They have special equipment but the rescue is often hazardous and may take many hours of exhausting work to achieve. It behoves all those who climb to respect these men and do their utmost to avoid imperilling the lives of those who are always ready and willing to give help when it is needed.

### CHALLENGE

When we think of the Matterhorn in the days of Whymper, the first man to climb it, and Everest in our own day, it may well be said that these climbs were attempted and finally achieved because they were of outstanding peaks and their "conquest"— would create prestige. Whymper's first comment on the Matterhorn are quite derogatory, but the mountain finally captured his imagination and he came to feel its uniqueness and beauty.

Men had explored and climbed in the Himalayas for some years before Everest was attempted, but when you look at these pictures you must admit that to a mountaineer it is a challenge that is difficult to ignore. A great deal of mountaineering went into the successful climbing of Everest and Sir John Hunt and Sir Edmund Hilary were the first to admit they climbed Everest on the shoulders of their predecessors. A short account from one of the earlier climbers I think will be of interest as an example of the effort and hazards that were experienced.

Somervell, a London surgeon who addressed this Society on 16th May 1923, his subject being "The Problems of Living at High Altitudes", was in 1924 one of five men who stood at 28,000 feet on Everest. He and Norton were fighting their way up towards the 28,000 feet mark, and they had reached the stage where they had to take ten breaths for every step and sit down to rest every five minutes. Their reconnoitering done, they turned to go back to their last camp. Norton was in front and it was getting dark, and they were not roped. Somervell had one of his fits of coughing from which he had been suffering and dislodged something which stuck in his throat so that he could not breathe, either in or out. He could make no sign to Norton who had gone on, so he sat down on the snow to die. Finally he made one great effort, pressing his chest with both hands, and the obstruction came up; he coughed up a little blood and was able to breathe more freely than he had done for several days. The obstruction was a slough of mucous membrane lining the larynx, the result of frostbite due to the rapid inhalation of very cold air for so many hours a day.

Both on snow and rock effort and concentration are required. Picture I was taken on a rock climb in Wales, and it shows the leader in a rock chimney using the technique for this ascent and you can see the attitude showing concentration and effort. A different technique is employed for a crack as distinct from a chimney, but this also requires absolute concentration.

When on a rock face, to get the maximum pressure on the holds with hands and feet, the aim should be to avoid hugging the rock. In Picture II is a very good example of this position, the feet especially are pushed into the foot holds.

Sometimes in descending a cliff or snow slope the only route is to negotiate an overhang. This can be done by the use of ropes but the problem is to get the last man down. An abseil is the answer. The rope is doubled around some belay above the overhang and then passed over the shoulder and under the thigh of the climber. When the climber has lowered himself to the rock beneath him the doubled rope can be pulled from the belay.

### REWARDS

On a mountain I have found that both the opportunity and the desire to stay quiet not infrequently happens, and occasionally one reaps a tremendous and unexpected reward. I would like to give you an account of an experience the memory of which has remained fresh with me all my life. It is necessary to tell you briefly the incidents that led up to this great moment. When I was in India four of us decided to go into Kashmir, not by the orthodox route, but by the pass at the eastern end of the valley, provided it was open, it being the middle of May. We arrived at the foot of the pass and engaged nineteen porters to carry our kit, the loads having to be light as the way was steep. We set off before it was light, still uncertain whether Kashmiris had yet crossed and made a track. In a short while I became impatient of the slow pace and the presence of porters on the rough track and went on up alone and in due course the party behind me were out of sight. The track was steep and rough but not too hard going until at about 9,000 feet it divided right and left. I turned right but in a short time the track appeared to peter out on to a precipice of five or six thousand feet. I turned back and took the left hand track; this soon became the route for an ice stream, and so I thought the only thing to do was to go straight up. The climbing at first was over the rocks that were not too difficult, and then towards the 10,000 foot level I came on to snow, and visibility was reduced to about sixty feet by cloud. I went over the snow slope and in due course found a hut—this I knew was for Kashmiris crossing the pass who might be caught in a blizzard. Not knowing in which direction the track over the pass was, if it existed at all, I made tracks from the hut north, south, east and west, finding my way back to the hut on my own tracks, but failing to find the Kashmiri track down into the valley. I decided I must be bolder, and reasoning that the track would be east of the hut made a much longer journey and came on to a narrow track in the snow going north and south. This must be the Kashmiri track. I followed downhill towards what I hoped was the Valley of Kashmir but the cloud still gave me very little visibility. After descending about 1,000 feet I saw a pinnacle of rock with a flat top and decided to rest a while. It was about 3.0 p.m. and I had been going since early morning without rest and without food. As I sat on my pinnacle I noticed the clouds were rising. I watched almost breathless with anticipation as they lifted like a curtain in an enormous theatre. Presently I saw below me my first view of the Vale of Kashmir. The river Jhelum wound its way like a silver snake along the valley and on each side were the flooded rice fields and the brilliant yellow of the saffron in full bloom. They looked like squares of gold and silver in the bright sunlight. The clouds continued to rise and soon before me across the valley were revealed the peaks of the main Himalayan range and among them that queen of mountains Nanga Parbat, the fifth highest mountain in the world. I have no idea how long I sat and drank in the scene and experienced utter peace. I must admit that later, distant calls and the arrival of the party with food was not unwelcome, but their reproaches fell on deaf ears and have been forgotten. That one memory filled my day and has remained with me ever since.

You, too, must have had your great moments; keep them and cherish them, they may stand you in good stead one day.

"Only a hill, earth set a little higher
Above the face of earth: a larger view,
Of little fields and roads: a little nigher
To clouds and silence: what is that to you?
Only a hill; but all of life to me,
Up there, between the sunset and the sea."

When you have been in general practice, especially in the country, for a long time, you establish a relationship with your patients which is unique. You may also establish close and enduring friendships among your patients. These friends will come to you with their troubles, which may not always be medical ones, but as their friend and physician you will help them with your advice and guidance. But this friendship cannot be entirely reciprocal. If you yourself should be in trouble, and perhaps pass through the dark night of sorrow, you may want to look for a friend for help and guidance, and you may find that there is no one to whom you can go. Your friends have always looked to you as their friend and physician, as a rock which is secure, on which they can rely; you therefore feel that if you go to them you make yourself vulnerable, that they will not look upon you as someone set apart who is absolutely secure, and you feel they may never have again that complete confidence in your help and guidance, because you have shown that you too have weaknesses. So you turn away and you feel, very, very lonely. Many of you will have found a solution to this, some in music, some perhaps in painting or even in drama. I count myself fortunate in being among those who have found consolation by having been alone on a mountain. "For there is much comfort on high hills and a great easing of the heart."

## Figures and Tables

**Figure f1:**
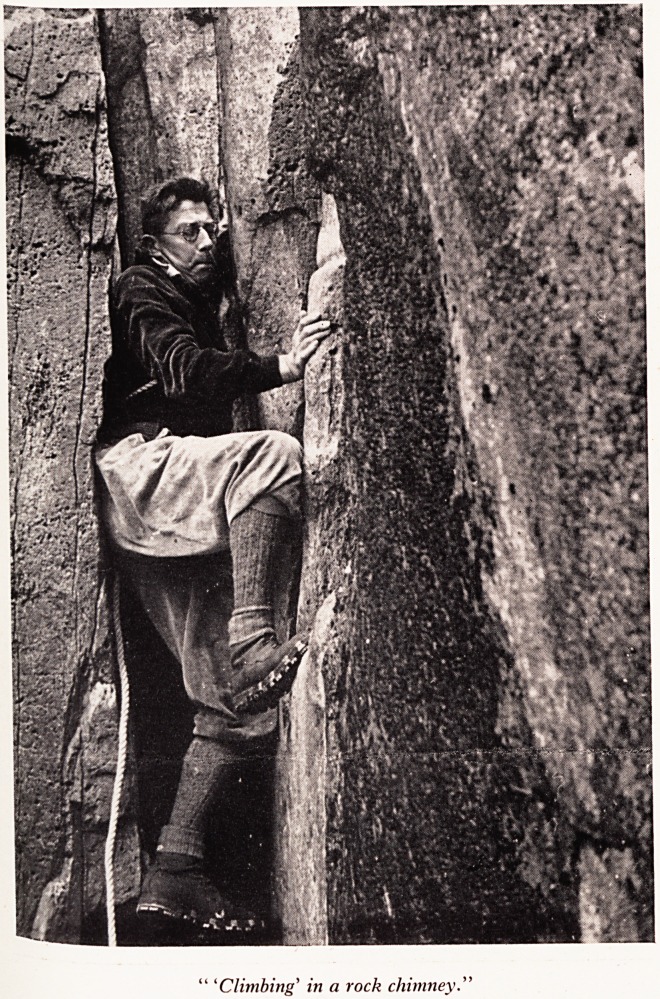


**Figure f2:**